# Based Upon Repeat Pattern (BURP): an algorithm to characterize the long-term evolution of *Staphylococcus aureus *populations based on *spa *polymorphisms

**DOI:** 10.1186/1471-2180-7-98

**Published:** 2007-10-29

**Authors:** Alexander Mellmann, Thomas Weniger, Christoph Berssenbrügge, Jörg Rothgänger, Michael Sammeth, Jens Stoye, Dag Harmsen

**Affiliations:** 1 Institute for Hygiene, University Hospital Münster, Münster, Germany; 2 Department of Periodontology, University Hospital Münster, Münster, Germany; 3 Ridom GmbH, Würzburg, Germany; 4 Center for Genomic Regulation, Barcelona, Spain; 5 Faculty of Technology, University Bielefeld, Bielefeld, Germany

## Abstract

**Background:**

For typing of *Staphylococcus aureus*, DNA sequencing of the repeat region of the protein A (*spa*) gene is a well established discriminatory method for outbreak investigations. Recently, it was hypothesized that this region also reflects long-term epidemiology. However, no automated and objective algorithm existed to cluster different repeat regions. In this study, the Based Upon Repeat Pattern (BURP) implementation that is a heuristic variant of the newly described EDSI algorithm was investigated to infer the clonal relatedness of different *spa *types.

For calibration of BURP parameters, 400 representative *S. aureus *strains with different *spa *types were characterized by MLST and clustered using eBURST as "gold standard" for their phylogeny. Typing concordance analysis between eBURST and BURP clustering (spa-CC) were performed using all possible BURP parameters to determine their optimal combination. BURP was subsequently evaluated with a strain collection reflecting the breadth of diversity of *S. aureus *(JCM 2002; 40:4544).

**Results:**

In total, the 400 strains exhibited 122 different MLST types. eBURST grouped them into 23 clonal complexes (CC; 354 isolates) and 33 singletons (46 isolates). BURP clustering of *spa *types using all possible parameter combinations and subsequent comparison with eBURST CCs resulted in concordances ranging from 8.2 to 96.2%. However, 96.2% concordance was reached only if *spa *types shorter than 8 repeats were excluded, which resulted in 37% excluded spa types. Therefore, the optimal combination of the BURP parameters was "exclude *spa *types shorter than 5 repeats" and "cluster *spa *types into spa-CC if cost distances are less than 4" exhibiting 95.3% concordance to eBURST. This algorithm identified 24 spa-CCs, 40 singletons, and excluded only 7.8% *spa *types. Analyzing the natural population with these parameters, the comparison of whole-genome micro-array groupings (at the level of 0.31 Pearson correlation index) and spa-CCs gave a concordance of 87.1%; BURP spa-CCs vs. manually grouped *spa *types resulted in 95.7% concordance.

**Conclusion:**

BURP is the first automated and objective tool to infer clonal relatedness from *spa *repeat regions. It is able to extract an evolutionary signal rather congruent to MLST and micro-array data.

## Background

*Staphylococcus aureus*, a human commensal living on the skin and mucosa, can cause a broad range of infections including endocarditis, septicemia, skin infections, soft tissue infections, and osteomyelitis. Moreover, *S. aureus *is the leading cause of nosocomial infections [1]. The application of several new genotypic typing methods gave many new insights into the epidemiology and population structure of *S. aureus *[2]. Recently, Koreen et al. investigated a collection of 36 *S. aureus *isolates (methicillin resistant and methicillin sensible *S. aureus*, MRSA and MSSA, respectively), which was recovered from 10 countries on four continents over a period of four decades as a representative of the breadth of diversity within *S. aureus *[3]. They used whole-genome micro-array analysis (comprising approximately 2,800 open reading frames) as typing reference to evaluate the capability of several typing techniques, among them partial *S. aureus *protein A (*spa*) gene sequencing. The *spa *repeat region consists of a variable number of 21–27 bp long repeats (VNTRs) varying in composition that result in different *spa *types.

Previously it was shown that *spa *typing is fast, discriminatory, and very reproducible [4,5]. It was hypothesized by Koreen and colleagues that by manual grouping of similar *spa *types this region contains evolutionary signals nearly comparable to whole-genome micro-array data [3]. Until recently, however, no automated and objective algorithm existed to cluster different repeat regions. The Based Upon Repeat Pattern (BURP) implementation that is a heuristic variant of the newly described EDSI algorithm [6], was investigated in this study to infer the clonal relatedness of different *spa *types. We first calibrated the BURP parameters using multilocus sequence typing (MLST) data from a representative strain collection as "gold standard" and then evaluated BURP using the Koreen et al. dataset.

## Methods

*S. aureus *strains (MRSA and MSSA) were used from our strain collection comprising 400 of the initial and most frequently to the SpaServer reported *spa *types [7]. From these strains, MLST sequence types (ST) were determined as previously [8]. STs that showed at least six of seven identical alleles were grouped into clonal complexes (CC) using eBURST [9]. BURP – as implemented in the StaphType software v. 1.5 (Ridom GmbH, Würzburg, Germany) – was used to cluster (spa-CC) *spa *types [10]. Repeat-duplication and -excision in addition to substitution and base-insertion and -deletion events were taken into account when the relatedness of different *spa *types was calculated. BURP offers two user-defined parameters that influence clustering: exclusion of *spa *types that are shorter than "x" repeats and the maximum number of costs "y" for clustering *spa *types into the same group. Short *spa *types can be excluded from further analysis because their information content is limited and no reliable evolutionary history can be inferred. The costs account for the "steps" of evolution between two different *spa *types, whereas the algorithm tries to minimize these steps ("parsimony assumption"). To find out the optimal combination of these two parameters, clustering of all possible combinations of both parameters (values: 1 to 10) was performed. A prerequisite was that the number of excluded *spa *types should be as low as possible and not exceed 10% of all investigated *spa *types. Subsequently, the typing concordance [11] between BURP and eBURST groupings were determined to elucidate the best parameter combination with the highest concordance on the one side and the lowest number of excluded *spa *types on the other. BURP calibrated in this manner was finally used to cluster the strains from the study of Koreen et al. [3].

## Results and discussion

**Figure 1:**
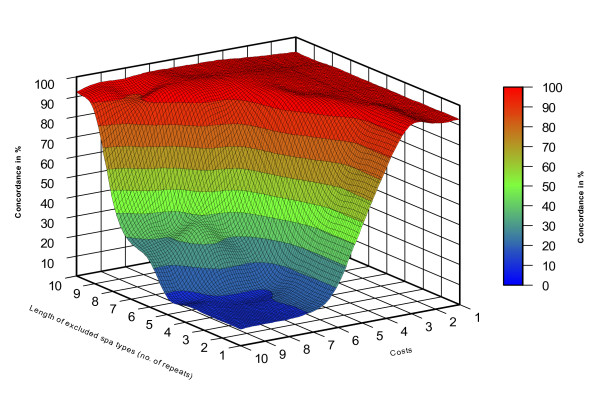
**Concordance analysis of eBURST and BURP clustering in dependence of all possible BURP parameters**. A surface curve displaying the dependence of concordance (in %) between eBURST MLST CCs and BURP spa-CCs applying all possible combinations of the BURP parameters "exclude *spa *types that are shorter than x repeats" and "*spa *types are clustered if costs are less or equal than y".

**Figure 2:**
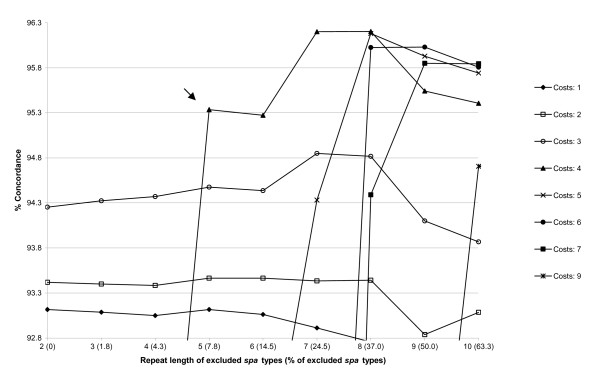
**High range of concordance between eBURST and BURP for optimal BURP calibration**. Graph showing curves for cost integers in the high concordance range. Curves labeled "Costs: 1 to 10" represent different cost values. For the curve with the overall highest concordance (Costs: 4) the first inflection point is marked (arrow) and corresponds to the first local optimum giving a good balance between concordance and percentage of excluded *spa *types.

**Figure 3:**
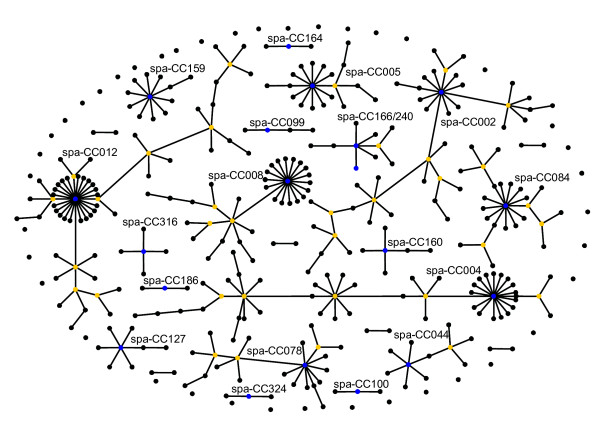
**Population snapshot of the 400 *S. aureus *strains after BURP grouping**. Population snapshot of the 400 *S. aureus *strains after grouping with the calibrated BURP ("exclude *spa *types that are shorter than 5 repeats" and "*spa *types are clustered if costs are less or equal than 4", 31 *spa *types were excluded). Clusters of linked isolates correspond to spa-CCs. Whereas eBURST uses the number of relatives (single locus variants, SLVs) to define founders and subfounders of groups, BURP sums up costs to define a founder-score for each *spa *type in a cluster. The *spa *type with the highest founder-score is defined founder of the cluster (blue color). Subfounders are the *spa *types with the second highest founder-score and are labeled in yellow. If two or more *spa *types exhibit the same highest founder-score, they are all colored in blue. For clarity, only the spa-CCs are labeled. Note that the spacing between linked *spa *types and between unlinked *spa *types and spa-CCs provides no information concerning the genetic distance between them.

In total, the 400 investigated strains exhibited 122 different STs. The eBURST algorithm clustered the STs into 23 CCs (354 isolates) and 33 singletons (46 isolates). BURP clustering of *spa *types using all possible parameter combinations and subsequent comparison with eBURST CCs resulted in concordances ranging from 8.2 to 96.2%. These concordances are illustrated in Figure 1 using the Visual-XSel 9.0 software (CRGraph, München, Germany). To determine the optimal combination between the BURP parameters, a graph showing the dependence of the concordance from the minimal repeat length of included *spa *types (vice versa the percentage of excluded *spa *types) for the different costs were drawn by MS Excel XP. The overall highest concordance (96.2%) lay on the cost value 4 curve (Figure 2). Analyzing this curve, the closest integer to the first inflection point – representing the first local maximum – was chosen as the optimal combination of the BURP parameters with "exclude *spa *types that are shorter than 5 repeats" and "*spa *types are clustered if costs are less or equal than 4". In this way a concordance of 95.3% could be achieved (Figure 2). Using these parameters, BURP clustered the 400 *spa *types into 24 spa-CCs and 40 singletons. Only 31 (7.8%) *spa *types were excluded by using these parameters. In contrast, analysis of ungrouped *spa *types vs. eBURST CCs resulted in 92.8% concordance, only. A population snapshot of the 369 included strains after BURP grouping is displayed in Figure 3. It shows clusters of linked *spa *types in spa-CCs, linked doublets, and individual unlinked *spa *types. In Table 1, exemplarily the spa-CC004, its *spa *types, corresponding STs, and CCs is shown. In general, a high concordance between BURP and eBURST clustering can be observed. Of the 50 *spa *types that were clustered in spa-CC004, only three *spa *types were grouped into another CC and another three were judged as singletons by MLST.

**Table 1:** Comparison of BURP and eBURST clustering results

**spa type**	**MLST ST**	**MLST CC**
t004, t015, t028, t029, t031, t033, t038, t040, t043, t049, t050, t061, t065, t069, t073, t077, t080, t095, t102, t116, t123, t124, t130, t141, t142, t157, t161, t204, t230, t247, t266, t277, t330, t331, t333, t340, t350, t361, t370, t371, t424	45	45
t180	53	45
t220	54	45
t295	278	45

t209	109	9
t133	254	239
t412	846	395
t302	625	singleton^a^
t397	842	singleton
t383	1008^b^	singleton

*spa *types, their corresponding MLST sequence types (ST), and clonal complexes (CC) of spa-CC004 are shown. ^a^no clonal complex was assigned for these singletons by eBURST analysis, ^b^this ST is preliminary named ST1008 and has the allelic profile 6, 5, 6, 6, 7, 17, 19.

Comparing whole-genome micro-array groupings (at the level of 0.31 Pearson correlation index) and spa-CCs of the 36 strains from the study of Koreen et al. using the calibrated BURP gave a concordance of 87.1% – that is in the same range as reported. BURP spa-CCs vs. manually grouped *spa *types resulted in 95.7% concordance.

The underlying alignment model of BURP takes repeat-duplication and -excision into account [6] – in contrast to widely-used multiple alignment strategies like ClustalW [12]. The proposed molecular mechanism of the evolution of such repeat regions is slipped-strand mispairing (SSM) during DNA duplication [13]. The presence of those evolutionary events within the *spa *repeat region was already detected in vivo, when sequential *S. aureus *isolates from long-term pulmonary *S. aureus *colonization/infection of cystic fibrosis patients were *spa *typed [14].

The high concordance of BURP spa-CCs in comparison to eBURST CCs using a diverse strain collection demonstrated that *spa *indeed contains long-term evolutionary signals. Recent comparisons between spa-CCs and PFGE clustering corroborated these findings [15,16]. In future, the integration of BURP into the already established early-warning system for MRSA-outbreaks based on *spa *typing will help to detect clonal diversification during extended outbreaks [17].

There are some limitations using spa-CCs for long-term analysis. First, the strains must be *spa*-typeable. Having typed more than 8,000 isolates, however, very few isolates (approximately 0.1%) were not typeable – probably due to mutations within the primer binding regions. Second, BURP analyses are limited to *spa *types that pass the parameter of a certain number of repeats. However, when analyzing the SpaServer content (accessed at 12^th ^September 2007) comprising 38,978 isolates with 2964 different *spa *types, only 204 (6.88%) of all *spa *types and 881 (2,26%) of all submitted isolates are effected, respectively. Finally, in very few instances, discrepancies can occur between *spa *and other typing methods as observed in this study and in two recent other publications [15,16]. These discrepancies are most probably due to recombinational events. Large chromosomal replacements that give rise to such typing incongruences have been experimentally documented for two STs previously [18].

## Conclusion

In summary, BURP is the first automated and objective tool to infer clonal relatedness from *spa *repeat regions. It is able to extract an evolutionary signal rather congruent to MLST and micro-array data.

## Abbreviations

BURP – Based Upon Repeat Pattern; CC – clonal complex; eBURST – electronic Based Upon Related Sequence Types; EDSI – excision and duplication of repeats, and substitution and indels of bases; MLST – multilocus sequence typing; MRSA – methicillin resistant *S. aureus*; MSSA – methicillin sensible *S. aureus*; PFGE – pulsed-field gel electrophoresis; *spa *– *S. aureus *protein A encoding gene; ST – sequence type; VNTR – variable number tandem repeats

## Competing interests

J. Rothgänger and D. Harmsen have declared a potential conflict of interest. J. Rothgänger and D. Harmsen are the developers of the Ridom StaphType software mentioned in the manuscript. The software is distributed and sold by the company Ridom GmbH that is partially owned by them. All other authors have declared that no competing interests exist.

## Authors' contributions

The project was coordinated by DH. AM and CB performed the laboratory work and data analysis. MS and JS developed the EDSI algorithm. TW and JR implemented BURP. AM, TW, and DH wrote the main part of the paper. All other authors gave useful comment on the analysis of data and text of the manuscript. All authors have read and approved the final version of the manuscript.
